# Steroid reduction‐resistant pulmonary involvement with Sweet's syndrome suspected of being vacuoles, E1 enzyme, X‐linked, autoinflammatory, somatic syndrome: A case report

**DOI:** 10.1002/rcr2.1288

**Published:** 2024-02-21

**Authors:** Yuki Amakusa, Tatsuro Suzuki, Masaya Takemura, Tetsuya Oguri

**Affiliations:** ^1^ Department of Respiratory Medicine Gamagori City Hospital Gamagori Japan; ^2^ Department of Respiratory Medicine, Allergy and Clinical Immunology Nagoya City University Graduate School of Medical Sciences Nagoya Japan; ^3^ Department of Education and Research Center for Community Medicine Nagoya City University Graduate School of Medical Sciences Nagoya Japan

**Keywords:** rare lung diseases, Sweet's syndrome, VEXAS syndrome

## Abstract

In cases of Sweet's syndrome with pulmonary involvement, fever of unknown origin, and macrocytic anaemia, VEXAS syndrome can be considered in the differential diagnosis. A 67‐year‐old man who was taking prednisolone for a fever of unknown origin and Sweet's syndrome was referred to us because of an abnormal chest shadow. Computed tomography revealed a nonfibrotic hypersensitivity pneumonitis‐like opacity, and blood test results indicated macrocytic anaemia. His pulmonary symptoms spontaneously improved but again exacerbated approximately 1 month later. Methylprednisolone pulse therapy improved his condition, but he had recurring fever flare and pulmonary involvement post‐treatment. A peripheral blood *UBA1* gene test planned at a specialized institution was not performed, making the diagnosis difficult. We attempted careful tapering of methylprednisolone, but his macrocytic anaemia led to pancytopenia and he unfortunately died of sepsis due to neutropenia.

## Graphical abstract

Few cases of lung lesions presenting with respiratory failure in Sweet's syndrome have been reported. We herein report a case of Sweet's syndrome in a patient suspected with VEXAS syndrome.

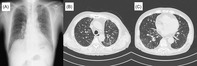

## INTRODUCTION

Few cases of lung lesions with respiratory failure in Sweet's syndrome have been reported.^1^ Differential diagnosis, including vacuoles, E1 enzyme, X‐linked, autoinflammatory, somatic (VEXAS) syndrome, is difficult at community hospitals. We report a case of Sweet's syndrome in a patient suspected with VEXAS syndrome and whose pulmonary lesions were resistant to steroid reduction.

## CASE REPORT

**FIGURE 1:**
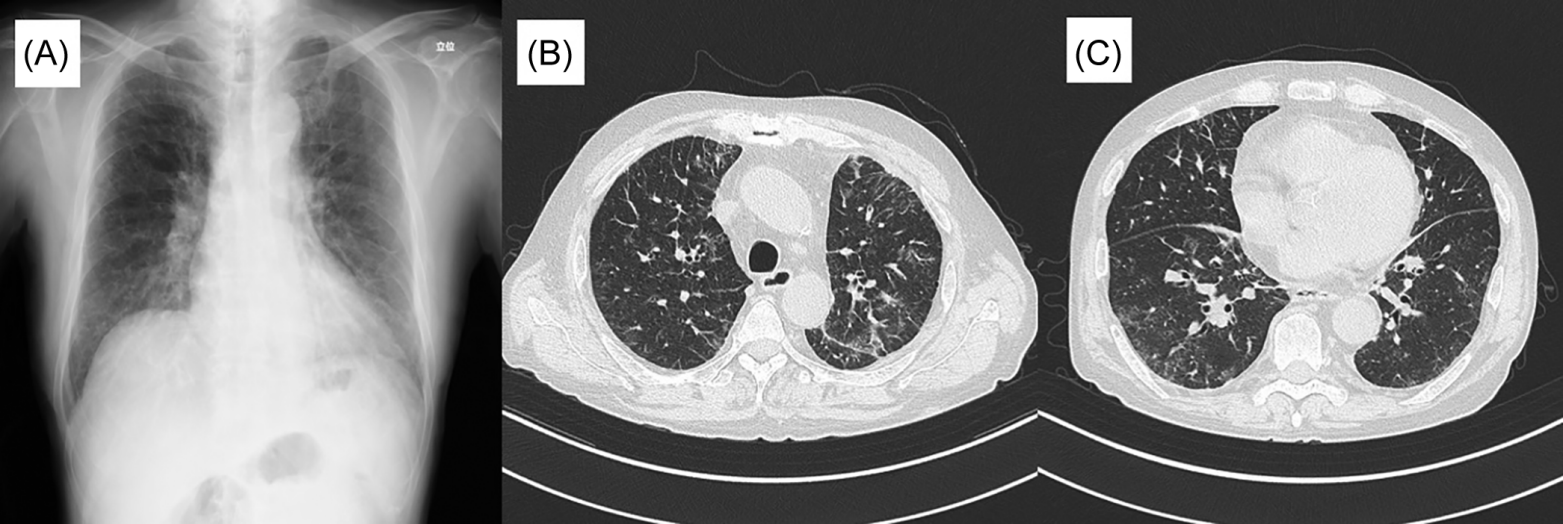
Imaging findings during the initial visit. (A) Chest x‐ray showing bilateral lung hypolucency and reticular opacities. (B and C) Chest computed tomography showing bilateral ground‐glass opacities, hypertrophic interlobular septa, mild mediastinal lymphadenopathy, and slight bilateral pleural effusions.

A 67‐year‐old man visited our hospital because of reticular shadows on chest X‐ray (Figure 1A). Five years earlier, he developed fever and painful erythema in the limbs and underwent skin biopsy, which revealed neutrophils and some lymphocytes present in the dermis, leading to diagnosis of Sweet's syndrome. Oral medications, including prednisolone, were started, which reduced his erythema. His fever recurred repeatedly during prednisolone dose reduction, but the disease could not be identified. Prednisolone dose was adjusted to 15–25 mg/day to limit his fever to ≤3 days/week.

**FIGURE 2:**
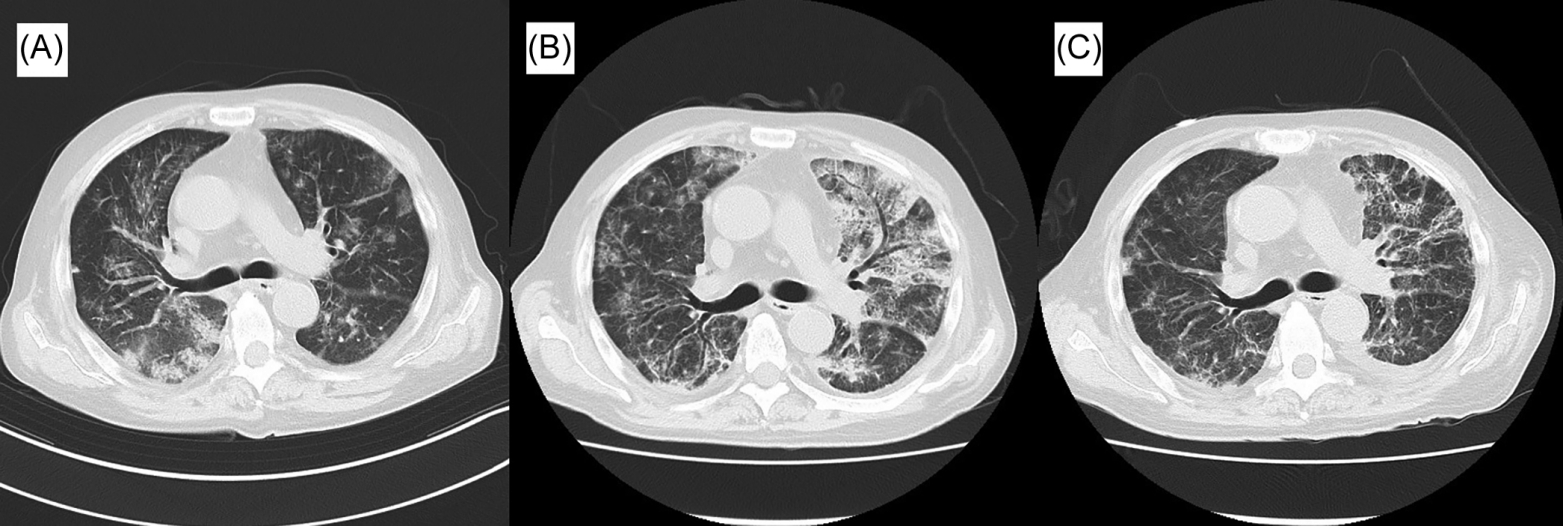
Progression of chest computed tomography (CT) imaging findings. (A) Chest CT scan obtained during hospitalization due to respiratory failure (hospital day 1). (B) Chest CT performed when the pulmonary involvement worsened during the administration of methylprednisolone at a dose of 40 mg/day after the initial methylprednisolone pulse therapy (hospitalization day 11). (C) Chest CT performed when the pulmonary involvement worsened during the administration of methylprednisolone at a dose of 80 mg/day after the second methylprednisolone pulse therapy (hospitalization day 18).

On physical examination, swelling of his ears or nose was not observed. Chest computed tomography (CT) revealed ground‐glass opacities and slight pleural effusion in both lungs, interlobular septa thickening, and mild mediastinal lymph node swelling (Figure 1B,C). CT findings were most similar to a nonfibrotic hypersensitivity pneumonitis pattern. His blood tests (Table 1) revealed elevated serum C‐reactive protein (CRP) levels and erythrocyte sedimentation rate. Imaging findings suggested hypersensitivity pneumonitis, but his serum KL‐6 level was normal. Serum βD glucan, cytomegalovirus pp65 antigen, *Trichosporon asahii* immunoglobulin G (IgG), and bird‐specific IgG antibodies as well as collagen disease and vasculitis markers were negative. Blood count evaluation revealed macrocytic anaemia, which was also present at previous blood tests of dermatology. However, vitamin B12 and folic acid were normal. Oral levofloxacin did not improve the lung shadows; therefore, bronchoscopy was performed. No findings of tracheochondritis, including mucosal redness and tracheal cartilage ring disappearance, were detected. Cell fraction and bronchoalveolar lavage fluid CD4/CD8 ratio were nonspecific, and *Pneumocystis jirovecii* DNA‐polymerase chain reaction findings were negative. Biopsies of the strong surrounding shadow tissues were performed, but no inflammation was observed. Post bronchoscopy, a *UBA1* gene variant test at a specialized institution was sought but they declined because their *UBA1* gene variant test limit had reached its maximum. We offered the patient a referral to a haematologist for macrocytic anaemia workup, including a bone marrow aspirate; however, he declined because he had no symptoms of anaemia. The patient continued living at home and his lung shadows improved slightly during follow‐up. Approximately 1 month later, his lung lesions worsened (Figure 2A) and respiratory failure occurred. He was hospitalized to avoid home antigens, but his oxygenation level dropped; thus, methylprednisolone pulse for 3 days was administered. The pulse therapy improved his lung shadows, fever, and serum CRP level, but his white blood cell count, which typically should increase with steroid administration, began to decrease, resulting in pancytopenia. After methylprednisolone treatment (40 mg/day), his fever, increased serum CRP levels, lung opacities (Figure 2B), and respiratory failure symptoms recurred. We administered the second methylprednisolone pulse and a cyclophosphamide pulse, and immediate improvement was again observed. However, even after administering 80 mg/day of methylprednisolone, fever, increased serum CRP level, pulmonary opacities (Figure 2C), and respiratory failure recurrence were noted. After the third methylprednisolone pulse course and post‐therapy with 125 mg/day of methylprednisolone, slight fever and slightly increased serum CRP levels were observed. Treatment with 125 mg/day methylprednisolone and 100 mg/day oral cyclosporin was continued, but his lung shadows and serum CRP levels remained same. When the patient's respiratory condition stabilized, he was scheduled for haematological consultation for transfusion‐dependent pancytopenia, suspected to be myelodysplastic syndrome, but he developed gram‐negative bacillus sepsis and febrile neutropenia before visit; his condition rapidly deteriorated, resulting in death on hospitalization day 46. His serum KL‐6, βD glucan, and cytomegalovirus pp65 antigen levels were normal throughout the disease course.

**TABLE 1:** Blood test results at the time of the initial visit.

Haematology		Serology		Biochemistory	
WBC	4900/μL	CRP	9.71 mg/dL	Iron	93 μg/dL
Neut.	79.0%	ESR	110 mm/h	Ferritin	715.8 mg/dL
Lymph.	15.0%	LD	182 U/L	Vitamin B12	190 pg/mL
Mono.	0.0%	KL‐6	260 U/mL	Folic acid	6.5 ng/mL
Eos.	3.0%	SP‐D	17.1 ng/mL	Copper	78 μg/dL
Baso.	0.0%	SP‐A	69.9 ng/mL		
AT‐lymph.	3.0%	β‐D glucan	7.1 pg/mL		
RBC	255 × 10^4^/μL	CMV pp65 antigen	(−)		
Hb	8.8 g/dL	BNP	24.3 pg/mL		
Hct	29.2%	IgG	1380 mg/dL		
MCV	114.5 fl	IgA	273 mg/dL		
PLT	37.4 × 10^4^/μL	IgM	145 mg/dL		
		IgG4	36.5 mg/dL		
		ANA	< 40×		
		PR3‐ANCA	< 1.0 U/mL		
		MPO‐ANCA	< 1.0 U/mL		
		ACPA	1.7 U/mL		
		Anti‐SSA Ab	(−)		
		ATA	(−)		
		Anti‐ARS Ab index	< 5.0		
		Bird specific antibody	(−)		
		anti‐*Trichosporon asahii* antibody	(−)		

Abbreviations: ACPA, anti‐cyclic citrullinated peptide antibody; ANA, antinuclear body; anti‐ARS Ab, anti‐aminoacyl‐tRNA synthetase antibody; Anti‐SSA Ab, anti‐Sjogren's‐syndrome‐related antigen A antibody; ATA, anti‐topoisomerase I antibody; CMV, cytomegalovirus; CRP, C‐reactive protein; ESR, erythrocyte sedimentation rate; MPO‐ANCA, myeloperoxidase‐anti‐neutrophil cytoplasmic antibody; PR3‐ANCA, proteinase‐3‐anti‐neutrophil cytoplasmic antibody.

## DISCUSSION

This patient was suspected of VEXAS syndrome, due to having fever of unknown origin, Sweet's syndrome, macrocytic anaemia and pulmonary involvement. At present, there is no diagnostic criterion for VEXAS syndrome, and diagnosis is currently based on the presence of a positive *UBA1* gene variant in peripheral blood leukocytes or bone marrow specimens, although only a few facilities can perform this testing. Therefore, some researchers have proposed diagnostic methods that do not include genetic testing. For example, Maeda et al. proposed a scoring system for VEXAS syndrome that uses clinical findings, age, cutaneous lesions, pulmonary involvement, chondritis, and macrocytic anaemia.^2^ Our patient had the relevant age and symptoms other than chondritis, and his score was 5. Maeda et al. stated that patients with a score of 5 probably have a *UBA1* variant and should certainly undergo *UBA1* genetic analysis. However, because the scoring system has not been confirmed by external validation, there are some concerns about its use.^3^

In a CT review of 45 cases of VEXAS syndrome with pulmonary involvement by Borie et al., bronchopneumonia‐like and organizing pneumonia‐like, as well as heart failure‐like shadows with unilateral pleural effusion and lymphadenopathy were reported. Moreover, there were some cases in which the imaging findings changed from heart failure‐like to bronchopneumonia‐like or organizing pneumonia‐like.^4^ In our patient, the pattern initially resembled nonfibrotic hypersensitivity pneumonitis; however, no studies to date have described the lung lesions of VEXAS syndrome as hypersensitivity pneumonitis patterns. Our patient's CT imaging after readmission revealed significant granular opacities and consolidation along the bronchi, which is a characteristic of VEXAS syndrome, suggesting a switch in imaging findings. To treat VEXAS syndrome, high‐dose steroids are used to effectively reduce inflammation, but dose reduction is often difficult, and conventional immunosuppressants are reportedly ineffective. In this case, the lung lesions recurred even after administering 80 mg/day of methylprednisolone following the second pulse. Thus, the presentation might not be a typical case of VEXAS syndrome, which is considered to respond to high‐dose corticosteroids. Janus kinase inhibitors can achieve steroid reduction in patients with VEXAS syndrome,^5^ but it is difficult to administer these therapies clinically because of the inability to perform gene variant diagnosis at community hospitals and insurance coverage.

In conclusion, when seeing a patient with steroid‐refractory Sweet's syndrome with pulmonary involvement, pulmonologists should consider VEXAS syndrome.

## AUTHOR CONTRIBUTIONS

Yuki Amakusa treated the patient, designed the study, and prepared the draft of the manuscript. Tatsuro Suzuki performed data collection. Masaya Takemura and Tetsuya Oguri critically revised the manuscript for important intellectual content. All authors reviewed the results, and read and approved the final version of the manuscript.

## CONFLICT OF INTEREST STATEMENT

None declared.

## ETHICS STATEMENT

The authors declare that appropriate written informed consent was obtained for the publication of this manuscript and accompanying images.

## Data Availability

The data that support the findings of this study are available from the corresponding author upon reasonable request.
